# Adherence to Antiretroviral Therapy Among Transgender and Gender Nonconforming People Living with HIV: Findings from the 2015 U.S. Trans Survey

**DOI:** 10.1089/trgh.2019.0050

**Published:** 2019-10-25

**Authors:** Michelle Teti, L.A. Bauerband, Claire Altman

**Affiliations:** ^1^ Department of Public Health, University of Missouri, Columbia, Missouri.; ^2^ Department of Health Sciences, University of Missouri, Columbia, Missouri.

**Keywords:** ART adherence, HIV/AIDS, national survey, transgender

## Abstract

**Purpose:** This article includes an analysis of medication adherence among transgender and gender nonconforming (TGNC) people living with HIV (PLWH) from the 2015 U.S. Trans Survey (USTS), the largest survey of TGNC people in the United States.

**Methods:** Using data from the USTS, our analytic sample included 162 TGNC PLWH who had been prescribed antiretroviral (ART). We grouped respondents by adherent/nonadherent and compared demographic characteristics and potential adherence risk factors.

**Results:** Approximately 65.8% of participants reported “taking ART as prescribed” all the time (61.0% trans women and 85.7% of trans men). Black TGNC PLWH were more likely to be nonadherent than whites or Hispanics. Adherent participants reported higher rates of home ownership. Nonadherent PLWH reported higher rates of verbal harassment, sex work, and homelessness in the past year, and lower rates of visiting an HIV doctor for care in the past year.

**Conclusions:** USTS respondents living with HIV reported lower adherence than in other nationally representative studies of this population in medical care. HIV rates are higher among black individuals, and reported adherence is lower, suggesting this is a high-priority population. Findings also suggest that unstable sociostructural conditions (homelessness, sex work, etc.) compromise medication adherence. Future practice directions include integrating HIV treatment plans into larger support services for TGNC PLWH.

## Introduction

Transgender and gender nonconforming (TGNC) people are at high risk for HIV infection and in urgent need of HIV prevention and care services.^1–5^ TGNC is an umbrella term for persons whose gender identity, expression, or behavior does not conform to that typically associated with the sex to which they were assigned at birth; including people who identify as transgender and gender nonbinary. Our study included respondents with diverse experiences and identities of gender; therefore, we will refer to them as TGNC. However, many studies we cite represented specific subgroups within the TGNC population, and wherever possible we have tried to use the specific language of the sample of studies referenced.^6^ The percentage of transgender people who were diagnosed with HIV in 2015 was three times the national average.^2^ Nearly 25% of all transgender women are living with HIV and more than one-half of black transgender women are living with HIV.^1^ A global review of HIV prevalence in 15 countries with available data indicated that transgender women have 49 times the odds of having HIV compared with general population.^4^ Although transgender men are generally less likely to acquire HIV than transgender women, their rates of infection are notably higher than the general population.^2,7^ In addition, given the dearth of HIV research about transgender men and the potential for HIV risk data collection categories to obscure transgender men under “heterosexual contact,” it is likely that their risks and HIV rates are greatly underreported.^2,7^

A complex combination of individual, community, and structural factors, including TGNC stigma and discrimination, contributes to the risk TGNC people face for HIV and compromise their ability to access and adhere to antiretroviral (ART) treatment once they have the virus.^2,4,8,9^ Such stigma and discrimination are associated with poor mental health, substance abuse, violence, lack of familial and social support, homelessness, and unemployment—all of which compromise HIV care.^2,9^ Additional barriers include TGNC people's limited access to and engagement in medical care, including HIV care,^10–12^ and lack of knowledge and sensitivity among health care providers to treat TGNC people and address their needs.^11,13^

Advances in HIV treatment, however, have created opportunities for people living with HIV (PLWH) to live longer and healthier lives. Adherence to ART therapy, for example, can decrease morbidity and mortality and eliminate HIV transmission.^14^ Thus, it is critical to the health of TGNC people with HIV (TGNC PLWH) and the future of the U.S. TGNC HIV epidemic, to understand and address the HIV medication adherence patterns of TGNC PLWH.

A limited existing body of research describes HIV medication adherence among transgender men and women. Analysis of data from the Medical Monitoring Project (MMP—a surveillance system supported by the Centers for Disease Control and Prevention that produces nationally representative data about adults living with HIV receiving medical care in the United States) identified basic adherence patterns. Among transgender men sampled in the MMP reporting current ART use, Lemons et al. found that 69% were currently virally suppressed, and 60% had sustained viral suppression for the past year.^3^ Mizuno et al. reported that 80.5% of trans women sampled in the MMP reporting current ART use self-reported adherence to their prescribed ART dose.^15^ Both studies described high rates of poverty, unmet service needs, depression, and substance use among transgender men and women. A few smaller community samples (*N*=22 to *N*=181) report lower ART adherence rates spanning from 55% to 68% among predominantly transgender women samples, although measurement of adherence varies across studies.^12,16–20^ Community studies of transgender populations report adherence barriers such as negative health care experiences^16^ and transphobia.^12^

The National HIV/AIDS Strategy for the United States calls for improving health outcomes and decreasing health disparities among transgender people.^21^ Meeting these goals necessitates better understanding of HIV adherence among this population,^18^ although existing national community samples are few.^12^ In this study we use the U.S. Transgender Survey (USTS) to explore factors associated with ART adherence among TGNC PLWH based on both the adherence literature and availability of variables in the USTS, including^22^ demographics, health and health care indicators, substance use, and recent negative experiences of discrimination and sexual assault.^22^

## Methods

### Data

The 2015 USTS is the largest survey of TGNC people in the U.S. Participants (*N*=27,715) include people ≥18 years who identified as trans, genderqueer, nonbinary, and other identities on the transgender identity spectrum. Recruitment efforts were multipronged and included outreach to trans people through >800 health organizations, health centers, LGBTQ groups, and online communities. These agencies shared information about the survey with their members through various in person and online communication channels. A recruitment advisory committee, survey-taking events, and cash prize drawings further assisted survey efforts. The USTS survey instrument was developed over the course of a year by a core team of researchers and advocates in collaboration with dozens of individuals with lived experience, advocacy and research engagement, and subject-matter expertise. Data collection procedures were approved by a full board review by the University of California—Los Angeles (UCLA) Institutional Review Board (IRB). The survey and consent procedures were administered online. The USTS was anonymous, so participants did not have to share any information that could identify them, such as their name.^22^ The current data analyses received IRB exempt approval from the authors' IRB.

### Analytic sample

Respondents were coded as living with HIV using two questions about HIV testing and most recent test result (*n*=179). Of the respondents who reported a positive test result, 17 had never been prescribed ART. Our analyses include the 162 respondents who reported being prescribed ART.

### Variables

#### Years since diagnosis

Participants were asked when their most recent HIV test was by month and year. We calculated the time, in months, between their reported last test and the time of the survey (August 2015) to estimate years since diagnosis.

#### Gender identity

Respondents had to answer their sex assigned at birth (male or female) to complete the survey. They were given the option of selecting all the gender identities that applied to them from a list of 25 (e.g., agender, butch, multigender, stud, and transsexual: full list available in report) with a write-in, but then they were asked to indicate which of the following options best described their current identity: cross-dresser, woman, man, trans woman (MTF), trans man (FTM), and nonbinary/genderqueer. Given the small sample size, and ability to generalize to other studies, we used the assigned sex question and the single selection of current identity to identify respondent gender identities. If respondents were assigned male at birth and identified as woman or trans woman we categorized them as trans women. If respondents were assigned female at birth and identified as man or trans man we categorized them as trans men. Regardless of assigned sex, anyone who selected cross-dresser (*n*=4) or nonbinary/genderqueer (*n*=20) were categorized as gender nonconforming and cross-dresser (there were not enough responses to separate).

#### Adherence

Respondents who reported being prescribed ART were asked if they were currently taking ART, and if they were taking ART as prescribed. Respondents who had been prescribed ART but reported that they were not currently taking ART were coded as nonadherent. Achieving HIV RNA suppression (<50 copies/mL) is contingent upon consistently high (>80%) ART adherence^23^; therefore, only respondents indicating taking ART such as “you're supposed to” all of the time were coded as adherent. Respondents who selected “never,” “rarely,” or “most of the time” were coded as nonadherent.

#### Demographics

Race was measured using a single-item question with racial/ethnic identity options. Included in the options were black/African American and white/European American. Respondents who identified as more than one race selected biracial/multiracial and completed follow-up selections to be able to indicate all their identities. For our analyses, sample size only allowed for specifically reporting white only, black only, and everyone else. Education was measured using reported highest level of school or degree completed and categorized as any level below GED or high school diploma as “less than high school”; “some college” as some college or a type of associate degree; and “bachelor's degree or higher” as bachelor's degree or degree assumed higher (i.e., some graduate work, master's degree, doctoral degree, or professional degree). Current living arrangements were categorized as living alone or with others in an owned house/apartment/condo; living alone or with others in a rented house/apartment/condo; or other living arrangements. Homelessness in the past 12 months indicates an affirmative response to a question regarding housing experiences. Income was measured with a single item asking about total before taxes income for the year 2014 with categorical options ranging from no income to >150,000. Health insurance dichotomously indicates whether or not they were currently covered by any health insurance or health/coverage plan (yes=1).

#### Sex work history

Sex work history was assessed asking about lifetime engagement in sex or sexual activity for money and engagement within the past year.

#### Health

Self-rated health was measured with a single question: “Would you say that your health in general is….” Response options ranged from poor (1) to excellent (5). Psychological distress was measured with the Kessler Psychological Distress Scale. The validated short-item scale is a six-item measure that asks about feelings in the past 30 days. Each feeling is measured on a scale of 0 (none of the time) to 4 (all of the time). Scores range from 0 to 24 with higher scores indicating greater psychological distress and scores >13 being considered “serious psychological distress.”^24^

#### Health care

Respondents report if they did not see a doctor in the past year due to cost (1=yes). Respondents also reported receiving care from a doctor for HIV in past 12 months.

#### Substance use

Heavy alcohol use was defined as respondents who reported consuming five or more drinks at least four times in the past 30 days. Illegal/illicit drug use was captured through a two-part question in which respondents first selected the type of substances used from a list that included an option for “Illegal or illicit drugs (such as cocaine, crack, heroin, LSD, and inhalants such as poppers or whippets).” A follow-up question was asked regarding whether this was used within the past 30 days, >30 days, or >12 months ago.

#### Past year negative experiences

Respondents reported whether they had been denied equal treatment in the past year for any reason (1=yes) or experienced verbal harassment for any reason in the past year (1=yes). Respondents reported whether they had experienced unwanted sexual contact in the past year (1=yes).

### Analysis

All analyses compared nonadherent and adherent individuals. *T*-tests (continuous measures) and chi-square tests (dichotomous and categorical) were used to assess significance of difference between the two groups on variables related to adherence. Table 1 lists means and group proportions for demographic, health indicators, and experiences within the past year by adherence.

**Table 1.** Comparisons of Nonadherent and Adherent HIV+ Individuals Across Demographics, Health-Related Indicators, and Recent Experiences (2015 U.S. Trans Survey)

	Nonadherent	Adherent^a^		Total
*N* (%)	55 (34)	107 (66)		162 (100)
	*M* (95% CI)	*M* (95% CI)	Cohen's *d* (95% CI)	*M* (95% CI)
Age	39.1 (36.1–42.0)	42.5 (40.4–44.5)	−0.32 (−0.64 to 0.01)	41.3 (39.6–43.0)
Years since diagnosed	6.6 (4.3–9.9)	7.5 (5.6–9.4)	−0.10 (−0.44 to 0.24)	7.2 (5.8–8.7)

	*n* (%)	*n* (%)	*χ*^2^	*n* (%)
Gender identity				
Transgender women	48 (87.3)	76 (71.0)	5.51	124 (76.5)
Transgender men	2 (3.6)	12 (11.2)		14 (8.6)
GQ/N/cross-dresser	5 (9.1)	19 (17.8)		24 (14.8)
Race				
White	21 (43.8)	55 (59.1)	7.36^*^	76 (53.9)
Black only	22 (45.8)	22 (23.7)		44 (31.2)
Latino/a/Hispanic only	5 (10.4)	16 (17.2)		21 (14.9)
Education				
Less than high school	6 (10.9)	8 (7.5)	4.04	14 (8.6)
High school	7 (12.7)	14 (13.1)		21 (13.0)
Some college	34 (61.8)	55 (51.4)		89 (54.9)
Bachelor's or more	8 (14.5)	30 (28.0)		38 (23.5)
Current living arrangements				
Own home	2 (4.2)	25 (24.5)	12.05^**^	27 (18)
Rent home	31 (64.6)	62 (60.8)		93 (62)
Other	15 (31.3)	15 (14.8)		30 (20)
Homeless in past 12 months	19 (34.5)	11 (10.2)	14.18^**^	30 (8.2)
Income				
<24,999	30 (65.2)	43 (43.9)	5.70	73 (50.7)
25,000–49,999	7 (15.2)	24 (24.5)		31 (21.5)
>50,000	9 (19.6)	31 (31.6)		40 (27.8)
Sex work				
Ever engage in sex work	38 (69.1)	63 (58.9)	1.63	101 (62.3)
Sex work past year	25 (45.5)	16 (15.0)	18.28^**^	41 (25.3)
Uninsured	4 (7.3)	12 (11.3)	0.66	16 (9.9)

	*M* (95% CI)	*M* (95% CI)	Cohen's *d* (95% CI)	*M* (95% CI)
Health				
Self-rated health	2.9 (2.6–3.1)	2.6 (2.4–2.8)	0.26 (−0.07 to 0.59)	2.7 (2.5–2.8)
Psychological distress	9.2 (7.6–10.8)	10.5 (7.4–13.6)	−0.10 (−0.42 to 0.23)	10.1 (8.0–12.2)

	*n* (%)	*n* (%)	*χ*^2^	*n* (%)
Health care in past 12 months				
Did not see a doctor due to costs	17 (31.5)	17 (16.2)	4.96^*^	34 (21.4)
HIV doctor visit	51 (92.7)	104 (97.2)	1.75	155 (95.7)
Substance use				
Heavy alcohol use	3 (5.6)	8 (7.6)	0.24	11 (6.9)
Ever used illegal/illicit drugs	35 (64.8)	53 (49.5)	3.38	96 (54.7)
Illegal/illicit drugs past year	23 (41.8)	20 (18.7)	9.96^**^	43 (26.5)
Negative experiences past 12 months				
Denied equal treatment for any reason	12 (21.8)	23 (21.7)	0.0	35 (21.7)
Verbally harassed for any reason	35 (63.6)	48 (44.9)	5.13^*^	83 (51.2)
Any unwanted sexual contact	12 (21.8)	17 (15.9)	0.87	29 (17.9)

Significance was tested using *t*-tests with continuous variables, and chi-squared for categorical variables.

^a^ Adherence indicated by selecting “currently taking ART as prescribed all of the time.”

^*^ *p*<0.05, ^**^p<0.01.

ART, antiretroviral.

## Results

Among the 162 HIV+ respondents, the average age was 41.3 (confidence interval [95% CI]: 39.6–43.0), 55 (34%) were nonadherent and 107 (66%) were adherent. The majority were transgender women (*n*=124, 76.5%), and most reported their race as white only (*n*=76, 53.9%) or black only (*n*=44, 31.2%). 50.7% (*n*=73) reported income <24,999, whereas 8.6% (*n*=14) indicated not completing high school (*n*=38, 23.5% reported a bachelor's or higher degree). Respondents reported an average of 7.2 years since being diagnosed with HIV (95% CI: 5.8–8.7), and 18% (*n*=27) currently owned a home. Independent *t*-tests for age, self-rated health, psychological distress, and years since HIV diagnosis by adherence were all nonsignificant. Table 1 reports the Cohen's d with the 95% CIs. The small sample sizes and large within-group variability yielded wide Cohen's d ranges.

We used chi-square analyses to compare demographics: gender, race, education, income, current living arrangements, and history of sex work. Given the small sample, we use three race categories: white, black only, and Latino/a/Hispanic only and find a significant difference, *χ*^2^(2)=7.36, *p*<0.05. There were a higher proportion of white and Latino/a/Hispanic individuals who were adherent. Current living arrangement was categorized into own home, rent home, or all other living arrangements. Individuals who were nonadherent had a higher proportion of “other” living conditions (roommate situations, living at home, etc.), whereas adherent individuals reported higher rates of owning a home *χ*^2^(2)=12.05, *p*<0.01. There was no difference in lifetime history of sex work, but individuals who were nonadherent reported higher rates of sex work within the past year *χ*^2^(2)=18.28, *p*<0.01. There were no other significant differences across demographic characteristics.

Health-related indicators, such as being uninsured, seeing an HIV doctor in the past 12 months, heavy alcohol use, and illegal/illicit drug use rates were compared between adherent and nonadherent individuals. There were no differences on these health-related indicators except for illegal/illicit drug use within the past year *χ*^2^(2)=9.96, *p*<0.01. Nonadherent individuals reported higher rates of illegal/illicit drug use than adherent individuals.

Experiences within the past year, including being denied equal treatment for any reason, verbally harassed for any reason, reported any unwanted sexual contact, homelessness, and did not see a doctor due to cost, were compared across adherence groups. People who were nonadherent were more likely to report verbal harassment, homelessness, and/or not seeing doctor due to cost than individuals who were adherent.

## Discussion

High incidence and prevalence of HIV among trans persons, the ability of ART to improve health and decrease HIV transmission, and little research on the experiences of TGNC PLWH relative to their cisgender counterparts underscore the importance of examining ART adherence among TGNC PLWH.^4,5,12^ To our knowledge, this is the first exploration of ART adherence using data from the USTS.^22^ Our findings that 66% of respondents taking ART took their meds all of the time is similar to rates found among other community samples of TGNC PLWH^12,18,20^ and lower than nationally representative samples of TGNC PLWH engaged in HIV care.^3,15^ The difference in community and nationally representative samples, highlighted again in this study by our findings, is important. One of the strengths of community samples such as the USTS is the sampling of PLWH across all levels of care, versus from PLHW who are connected to health care. Those not in care, such as those reached by the USTS, may provide a more realistic picture of community ART adherence levels and needs.

Direct comparisons across samples are complicated due to the varied ways of measuring and understanding adherence among TGNC PLWH. For example, in our study adherence is coded as “taking ART as prescribed all of the time.” However, Mizuno et al. measured “self-reported adherence to prescribed ART dose.”^15^ Among community samples, in a study about dual hormone/ART use, Braun et al. measured adherence as “taken differently than prescribed due to drug (hormone) interacting concern”^20^; Reisner et al. defined adherence by asking youth about the last 7 days taking HIV medications, one or more doses missed were categorized as not fully adherent days.^12^ There are pros and cons to each measurement approach, but lack of consistency prevents meaningful comparisons. Nonetheless, our findings provide empirical evidence of TGNC PLWH as adherent but with much room for improvement. This is important given the critical role that ART adherence plays in promoting the health of PLWH and preventing HIV transmission.

Our findings indicate that vulnerable TGNC PLWH are also the most at risk of poor adherence. For instance, racial/ethnic minority TGNC PLWH reported lower adherence than white TGNC PLWH. This finding supports existing intersectional research that racial/ethnic minority trans people report poorer health and social outcomes than white trans people,^25,26^ and that among PLWH overall, racial/ethnic minorities report lower rates of medication adherence and viral suppression than white PLWH.^27^ Such findings are likely related to intersecting forms of discrimination such as HIV, trans, and racial discrimination.^26,27^

Other factors affecting adherence were related to social instability and included homelessness and “alternative” (not renting or owning a home) housing, sex work, and drug use in the past year, not being able to access care because of finances, and experiencing verbal harassment. Some of these factors such as compromised access to care^15^ have been supported by existing adherence research with TGNC PLWH, and among PLWH overall, including homelessness,^28^ sex work,^29^ and drug use.^28,30^ As with adherence, different measures across studies may account for variation in risk, although sociostructural vulnerability is a common theme. In this study, only sex work highlights that current instability in people's lives is a barrier to their health, but suggests that lifetime experiences with these challenges may not affect current health patterns, and that change toward healthier patterns is possible. Identifying the most at risk TGNC PLWH is critical to directing cost-effective future interventions.

This study has important implications for future research and practice. Our study is among the few to describe adherence among a national community sample of trans people. Community samples are important sources of information as they may include individuals who may not be linked to HIV care, as in the existing national databases. Understanding the perspectives of PLWH who are not engaged in care is critical to increasing ART adherence and decreasing HIV transmission.^21^

Advancing adherence research among TGNC PLWH also requires consistent measures of adherence to allow comparisons across study designs. Thus, consensus and collaboration among researchers may be needed to create and implement more uniform measures of engagement in the care continuum. Future collections of the USTS could consider additional and more specific questions to expand upon current HIV adherence data. Most of the existing research among TGNC PLWH has been done with trans women. This is understandable given their increased risk, yet trans men are also infected with HIV at higher rates than the general population.^2^ And research on health in general among trans and nonbinary folks indicates poorer health outcomes among nonbinary subgroups.^31^ Thus, additional survey recruitment and research on trans men and nonbinary people's experiences with HIV and adherence are critical. Also important in future research is understanding how TGNC PLWH navigate all of their health challenges and what they say they need to adhere to medications, a contribution future qualitative research could make.

Practice-wise, our findings indicate that life instability (e.g., daily barriers, challenges, and changing circumstances that contribute to life stress and chaos) (citation withheld) is associated with nonadherence. Thus, findings among TGNC PLWH mirror those of PLWH overall and indicate that complex behaviors such as addiction or sex work combined with social challenges such as homelessness and violence, contribute to poor adherence.^32–34^ Few prevention interventions exist for trans people with HIV.^4^ More are needed to address adherence information and skills, but that also help people cope with their greater life challenges that interfere with their health. Community-informed interventions, peer interventions, and structural interventions hold promise.^5^

Although our research provides new insights, it is not without limitations. First, our measurement of adherence was constrained by the question used in the USTS that limits comparability with other studies. Second, the analytical sample size was small and Cohen's *d* intervals were large. Finally, this sample of trans men and gender nonbinary people was too small to draw specific conclusions.

## Conclusion

These results provide a picture of adherence among a community sample of TGNC PLWH. These findings contribute more information about trans people's medication adherence patterns and directions for future research and interventions. Meeting the U.S. HIV Strategy's goals of decreasing HIV among trans people requires a better understanding of challenges and strengths related to medication adherence.

## Abbreviations Used

ART: antiretroviral
IRB: Institutional Review Board
MMP: Medical Monitoring Project
PLWH: people living with HIV
TGNC: transgender and gender nonconforming
UCLA: University of California—Los Angeles
USTS: U.S. Trans Survey
